# Determination of drug-related problems among type 2 diabetes outpatients in a hospital in Vietnam: A cross-sectional study

**DOI:** 10.1371/journal.pone.0289825

**Published:** 2023-08-23

**Authors:** Duong Thi Ly Huong, Nguyen Thanh Hang, Nguyen Khanh Ly, Nguyen Hong Nhat, Nguyen Thi Lan Huong, Le Thi Phuong Hue, Dang Thi Lan Anh, Bui Thi Kim Dung, Phung Minh Phuong, Luong Thuy Lan, Truong Thanh Tung, Nguyen Ngoc Hieu, Ngo Hai Ly

**Affiliations:** 1 Faculty of Pharmacy, PHENIKAA University, Ha Dong, Hanoi, Vietnam; 2 Thanh Nhan Hospital, Hai Ba Trung, Hanoi, Vietnam; Nova Southeastern University / Yarmouk University, UNITED STATES

## Abstract

**Introduction:**

Drug-related problems (DRPs) are common in clinical practice and occur at all stages of the medication process. The major factor contributing to DRPs is prescription, although patients’ poor adherence to treatment is also a significant factor. This study evaluated type 2 diabetes outpatients in a hospital in Vietnam for drug-related problems (DRPs) and related variables.

**Methods:**

A cross-sectional descriptive study was conducted on 495 outpatients who met the criteria and 157 people agreed to participate in the interview. Medication order review and medication adherence review were used to identify DRPs. The types of DRP were based on the Pharmaceutical Care Network Europe (PCNE) categories version 9.0. The identification and assessment DRPs were carried out by clinical pharmacists and get agreed upon by physicians who had not directly prescribed patients who participated in the study.

**Results:**

A total of 762 DRPs were identified via prescribing review process, the average number of DRP on each prescription was 1.54±1.07, while 412 DRPs were determined through patient interviewing. The most frequent DRPs were “ADR (Adverse Drug Reaction) occurring” (68.8%). The main causes were “patient is unable to understand instructions properly” or “patient is not properly instructed”, “patient stores insulin inappropriately”, “patient decides to use unnecessary drugs” and “patient intentionally uses/takes less drug than prescribed or does not take the drug at all for whatever reason” which accounted for 65.0%, 41.4%, 38.2%, and 28.7%, respectively. From the prescribing review, the most observed DRPs were “Inappropriate drug according to guidelines/formulary” and “No or incomplete drug treatment in spite of existing indication”, accounting for 45.0% and 42.9%, respectively. There was a significant association between age (OR 3.38, 95% CI: 1.01–11.30), duration of diabetes (OR 3.61, 95%CI: 1.11–11.74), presence of comorbidity (OR 5.31, 95%CI: 1.97–14.30), polypharmacy (OR: 2.95, 95%CI: 1.01–8.72) and DRPs. In patients, poor knowledge of antidiabetic agents was the main reason to lack adherence and occurring ADR (OR 2.73, 95%CI: 1.32–5.66, p = 0.007 and OR 2.49, 95%CI: 1.54–4.03, p = 0.001 respectively).

**Conclusion:**

DRPs occurred in the prescribing stage and relating to patient’s behavior of drug administration was high. Clear identification of DRPs and the associated factors are essential for building the intervention process to improve effectiveness and safety in the treatment of type 2 diabetes mellitus patients.

## Introduction

A drug-related problem (DRP) is an event or circumstance involving drug therapy that actually or potentially interferes with desired health outcomes [1]. It has become a major challenge for healthcare systems due to their clinical and economic impact. The estimated annual cost of drug-related morbidity and mortality in the US was $528.4 billion, equivalent to 16% of total healthcare expenditures in 2016 [2]. Fortunately, half of them are potentially preventable [3]. Pharmacists play an important role in reducing DRPs by identifying and addressing them through a systematic medication review process. A study in Japan found that the potential cost savings from 2376 pharmaceutical interventions for 1678 drugs amounted to $2,657,820 with an average cost savings of $1511.0 per case of up to USD 2,657,820 [4].

The incidence of DRPs varied among studies, ranging from 8.54 to 99.16% and the average number of DRPs per patient ranged from 0.58 to 7.2 [5]. Treatment problems leading to DRP were mainly classified into two primary domains (effectiveness and safety), being treatment safety the most frequent problem [5]. The causes of DRPs were mainly in the prescribing section, including “drug selection” and “dose selection”, while patients’ poor adherence in the use section was also an important cause of DRPs [5]. Comorbidities and polypharmacy were considered as main factors associated with the occurrence of drug therapy problems [6–8].

Diabetes is a frequent metabolic condition. According to the International Diabetes Federation, 537 million persons (20–79 years) worldwide will have diabetes in 2021, with the figure expected to rise to 643 million by 2030 and 783 million by 2045 [9]. About 6.7 million people around the world have died from diabetes, and the medical costs of the disease are no less than $966 billion by 2021 [9]. Type 2 diabetes is the most common type of diabetes, accounting for over 90% of all diabetes worldwide. Management of type 2 diabetes (T2DM) is challenging because most patients are elderly with comorbidities, taking multiple medications, which can lead to medication errors.

There have been several studies on drug-related problems in diabetes [10–16]. Tadesse Sheleme (2021) determined that suboptimal drug therapy and untreated symptoms or indications were the most commonly identified drug-related problems. Diabetes duration of more than 7 years and the presence of comorbidities were predictors of drug-related problems [6]. Yaschilal Muche Belayneh (2021) exhibited the three most common DRPs in diabetes patients: additional drug therapy, non-compliance, and unnecessary drug therapy. Additionally, age of more than 45 years, presence of comorbidity, and emergency visit in the last one year were significantly associated with the occurrence of drug-related problems [17].

DRPs can occur at all medication usage steps, from prescribing to the dispensing stage [18]. Medication review (MR) was defined as a “structured evaluation of patient’s medicines with the aim of optimizing medicines uses and improving health outcomes” [19]. This entails detecting drug-related problems and recommending interventions [20]. Medication review is classified into 3 levels of review: 1) Medication order review: addresses issues relating to the prescription or medicines; the patient does not need to be present, nor access to full notes; 2) Medication adherence review: addresses issues relating to the patient’s medicine-taking behavior; 3) Clinical medication review: addresses issues relating to the patient’s use of medicines in the context of their clinical condition [21]. This was the first study in Vietnam to apply the two levels of medication review to identify DRP in the prescription stage and behavior of patient taking medicine. The purpose of this study was to clarify DRPs and associated factors through prescription review and adherence review in type 2 diabetes patients.

## Materials and methods

### Study design, setting, and population

A cross-sectional analysis of patients receiving diabetes care at Thanh Nhan Hospital between December 12, 2022, and January 12, 2023, satisfied the following inclusion and exclusion criteria:

#### Inclusion criteria

In order to participate in the study, participants must be at least 18 years old, have type 2 diabetes, be getting outpatient treatment, have fasting blood sugar (FBG) and glycated hemoglobin (HbA1C) tests, be taking diabetes medication, and have obtained consent.

#### Exclusion criteria

Pregnant women and those who experienced an emergency requiring hospitalization are excluded.

All patients who satisfied the aforementioned requirements had their prescriptions gathered in templates. Patients who agreed to be interviewed would be questioned in accordance with a questionnaire that the study team had created.

The study involved a group of five pharmacists and two endocrinologists. Prior to gathering data, the group had discussed, developed, and reached consensus on various scales for recognizing and assessing DRPs, such as a list of criteria for identifying drug selection that is not in accordance with recommendations. To assess the correctness of prescriptions, we created individual medication monographs with details on dose, administration, contraindications, etc. The survey groups were made up of students who had undergone in-depth training in the study, data gathering methods, and patient interviews.

After collecting data for a short while, we used our experiences to finish the process of gathering, evaluating, and creating templates and toolkits for assessment. To assess DRP, a pharmacist must fully understand the study and have obtained the necessary training in the evaluation techniques. Before the final findings were made public, the list of DRPs was assessed and analyzed by two endocrinologists with experience in diabetes mellitus who were not involved in any of the prescriptions acquired throughout the study.

The study followed Helsinki’s rules of ethics in medical research. The project was approved by the Ethics Committee of Vietnam National University, Hanoi (Board number: IRB-VN 01016).

### The sample size

Based on a systematic review of DRPs in Primary Health Care [5], the most common type of DRP was”treatment safety” which accounts for 42%, we calculated the sample size of 375 and above was suitable for identifying the expected proportion with 5% absolute precision and 95% confidence. This sample size was estimated following a software at https://statulator.com/SampleSize/ss1P.html

### Study variables

Dependent variables were DRPs that coded following the PCNE V9.0 classification system. Factors considered affecting DRP include the age, sex, duration of T2DM, polypharmacy, comorbidity and therapeutic group use. Because the study design was cross-sectional at a point in time, not the whole DRPs were assessed in this study. The list of DRPs assessed and addressed to evaluate was shown in Table 1.

**Table 1:** List of assessed DRPs according to PCNE V9.0 classification system and address to collect data.

	Code V9.1	Primary domains	Assessment/Address of data
**Problems (also potential)**	P1	Treatment effectiveness	*NA (because no agreement of evaluation effectiveness was achieved)
	P2	Treatment safety	Yes (patient interview and prescribing review)
	P3	Other	
**Causes**	C1	Drug selection	Yes (prescribing review)
	C2	Drug form	Yes (prescribing review)
	C3	Dose selection	Yes (prescribing review)
	C4	Treatment duration	*NA (because the study was a cross-over description)
	C5	Dispensing	*NA (because it is out of observation at this time)
	C6	Drug use process	*NA (because the patients who participated in this study were outpatients. They self-administration drugs without support from a health professional or carer
	C7	Patient related	Yes (Patient interview)
	C8	Patient transfer related	*NA (because no observation in the patient transfer was applied in this study)
	C8	Other	
**Planned Interventions**	I0	No intervention	*NA
	I1 I2 I3 I4	At prescriber level At patient level At drug level Other	*NA
**Intervention Acceptance**	A1 A2 A3	Intervention accepted Intervention not accepted Other	*NA
**Status of the DRP**	O0 O1 O2 O3	Problem status unknown Problem solved Problem partially solved Problem not solved	*NA

*NA: Not application

In patient: non-adherence behavior was identified through a questionnaire that detected DRPs coded C7.1, C7.2, C7.3, C7.4, C7.5, C7.7 by PCNE V9.0, described respectively as patient intentionally uses/takes less drug than prescribed or does not take the drug at all for whatever reason (C7.1), patient uses/takes more drug than prescribed (C7.2), patient abuses drug (unregulated overuse) (C7.3), patient decides to use unnecessary drugs (C7.4), patient takes food that interacts (C7.5), inappropriate timing or dosing intervals (C7.7). Patient’s knowledge was assessed through questionnaire that identified the causes of misuse drugs (the patient did not know that were wrong and had not received an appropriate guide from health workers), and DRPs coded C7.6 (patient stored drug inappropriately), C7.10 (patient unable to understand instructions properly).

### Data analysis

Descriptive statistics was used to analyze data. DRPs were shown as number and percentage. Continuous variables, such as HbA1C, FBG, and lipid profiles were described as Mean ± SD. A logistic regression model was used to identify the factors associated with DRP. Odds ratios (OR) and 95% confidence intervals (CI) were calculated. Variables included in the logistic regression models were age, sex, history of the disease, comorbidity and polypharmacy. The patient’s knowledge and adherence behavior were analyzed to determine the relation to occurring ADR. The analyses were carried out using SPSS for Windows, version 22.0. The significance level was set at 0.05.

## Results

### Demographic characteristics

**Fig 1:**
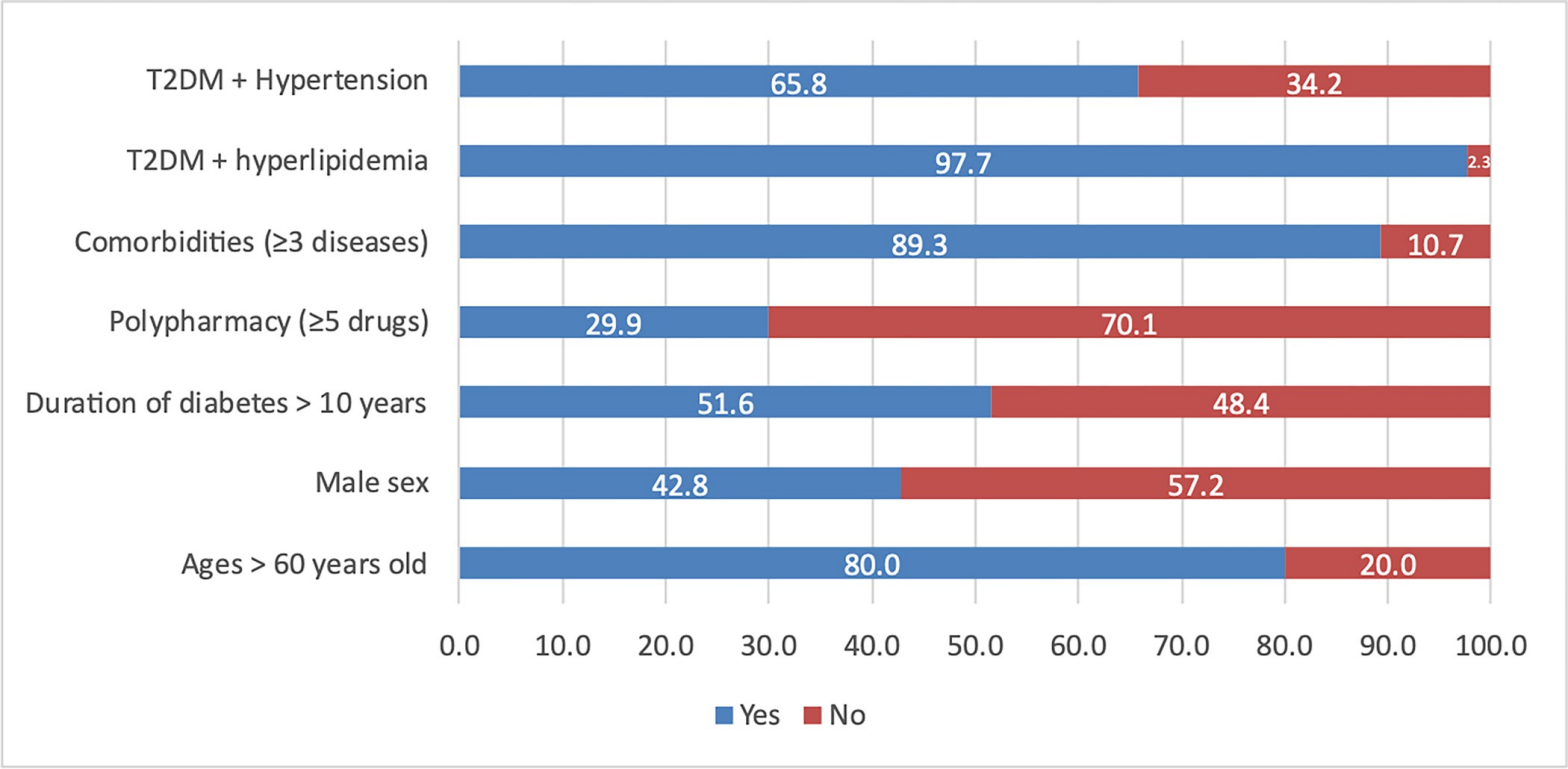
Percentage of patients with demographic characteristics.

A total of 495 T2DM patients fulfilled the inclusion and exclusion criteria for the study, while only 157 individuals (accounting for 31.72%) accepted the interview. The mean age of patients was 67.18 ± 9.88 years old, with the minimum and maximum years of age being 18 and 94, respectively. Most of the people (80%) were older than 60 years old. Females (57.2%) was higher than male (42.8%). The median time of diabetes was 10 years with a minimum and maximum year of 0 and 33, respectively. There was a new case of diabetes in this study. The majority of patients (97.7%) were diagnosed with both diabetes and hyperlipidemia, while 65.8% of patients had diabetes and hypertension concomitantly. The proportion of comorbidity in this study is rather high, with 89.3% of patients being diagnosed with three or more diseases and only two people got one disease. The average number of drug described among the population was 3.92±1.28 drugs. 148 patients (29.9%) received polypharmacy with five or more drugs concurrently per prescription (Table 2 and Fig 1).

**Table 2:** Demographic characteristics of participants.

Variables		n(%)	Mean ± SD (Mode)	Min—Max
**Ages**	≤ 60 years old	99/495 (20.0%)	67.18 ± 9.88	18–94
	> 60 years old	396/495 (80.0%)		
**Sex**	Female	283/495 (57.2%)		
	Male	212/495 (42.8%)		
**Duration of diabetes**	< 10 years	76/157 (48.4%)	Median: 10 years	0–33
	≥ 10 years	81/157 (51.6%)		
**Comorbidities**	Diabetes (T2DM) only	495/495 (100%)	3.88 ±1.18 (mode: 3)	1–8
	T2DM + hyperlipidemia	476/487 (97.7%)		
	T2DM + Hypertension	273/415 (65.8%)		
	No comorbidities (<3 diseases)	53/495 (10.7%)		
	Comorbidities (≥3 diseases)	442/495 (89.3%)		
**Polypharmacy**	No (<5 drugs)	347/495 (70.1)	3.92±1.28 (mode: 4)	1–7
	Yes (≥5 drugs)	148/495 (29.9)		

### Laboratory examination

The laboratory parameters of the participant were described in Table 3. To measure renal function, the Modification of Diet in Renal Disease (MDRD) formula was applied, and an estimate of glomerular filtration rate (eGFR) was calculated as follows:

$$eGFR=186\;x\;{\left(sCr\right)}^{‐1.154}\;x\;{\left(age\right)}^{‐0.203}\;x\;0.742\;(for\;females).$$

(Abbreviations and Units: eGFR, estimated glomerular filtration rate, mL/min/1.73 m^2^; Scr, serum creatinine, mg/dL; age, years)

**Table 3:** Laboratory parameters of participants.

Parameters	Values	n(%)	Mean ± SD
FBG (mmol/L)	≤ 7.2	211/495 (42.6%)	8.21 ± 2.63
	7.3–16.6	275/495 (55.6%)	
	> 16.6	9/495 (1.8%)	
HbA1C (%)	<7	187/495 (37.8%)	7.71 ± 1.53
	7–9	213/495 (43.0%)	
	> 9	95/495 (19.2%)	
CHO (mmol/L)	<5.2	240/487 (49.3%)	5.31 ± 1.17
	≥5.2	247/487 (50.7%)	
LDL (mmol/L)	<1.8	12/472 (2.5%)	3.41 ±0.96
	≥1.8–2.6	76/472 (16.1%)	
	≥2.6 - <3.6	198/472 (41.9%)	
	≥3.6 - <5.2	171/472 (36.2%)	
	≥5.2	15/472 (3.2%)	
TG (mmol/L)	<1.7	171/486 (35.2%)	2.45 ± 1.47
	≥1.7–2.3	117/486 (24.1%)	
	>2.3	198/486 (40.7%)	
HDL (mmol/L)	Low	62/129 (47.7%)	1.23 ± 0.27
	High	68/129 (52.3%)	
eGFR (mL/min/1.73m^2^)	>90	33/286 (11.5%)	72.43 ± 17.28
	60–89	190/286 (66.5)	
	45–59	45/286 (15.7%)	
	30–44	14/286 (4.9%)	
	15–29	4/286 (1.4%)	

FBG (fasting blood glucose), CHO (Cholesterol), LDL (low-density lipoprotein), TG (triglyceride), HDL (high-density lipoprotein), eGFR (estimated Glomerular Filtration Rate)

In Table 3, we can see that the number of patients who had controlled blood glucose (HbA1C<7%) is estimated to be 37.8%. There was a high proportion of co-hyperlipidemia in people with high cholesterol (≥5.2 mmol/L), LDL (≥2.6 mmol/L), TG (≥ 1.7 mmol/L) levels: 50.7%, 81.3%, and 64.8%, respectively. Only 26.1% and 57.8% of patients were tested for HDL and eGFR; among them, nearly half of the patients had low HDL, and the eGFR values of four people (0.4%) were less than 30 mL/minute/1.73m^2^. These are important preclinical parameters for prognosis assessment, and dose adjustments should be checked more frequently.

### Medication used in T2DM patients

100% of T2DM patients were prescribed antidiabetic agents, but only 83.6% and 36.4% of patients, respectively, were prescribed antihypertensive and antihyperlipidemic medications (Table 4). These untreated cases significantly contributed to DRP code C1.5 “No or incomplete drug treatment despite an existing indication”.

**Table 4:** Drugs prescribed in T2DM patients.

Categories	Number of patients diagnosed (n)	Number of patients treated (n,%)
Antidiabetes	495	495 (100%)
Antihypertension	273	229 (83.6%)
Anti-hyperlipidemia	476	174 (36.4%)

Among antidiabetic agents, the most frequent drugs used were metformin (80.8%, including plain and combination with sulfonylurea or DPP4 inhibitors), then sulfonylurea (59.4%) and insulin (36.4%). For antidiabetic regimens, the two drug combinations metformin and sulfonylurea, Metformin and Insulin were the most common (41.6% and 12.9%, respectively) (Table 5).

**Table 5:** Antidiabetic drugs used in T2DM patients.

Categories	Medicines	n(%)
Types of antidiabetic drugs	• Insulin	180 (36.4)
	• Metformin	400 (80.8)
	• Sulfonylurea	294 (59.4)
	• SGLT2 inhibitors	14 (2.8)
Antidiabetic regimens	Monotherapeutic:	91 (18.4)
	• Insulin	37 (7.5)
	• Metformin	27 (5.5)
	• Sulfonylurea (SU)	27 (5.5)
	Two drug combinations:	302 (61.0)
	• Metformin + Sulfonylure	206 (41.6)
	• Metformin + Insulin	64 (12.9)
	• Two insulin forms	23 (4.6)
	• Insulin + Sulfonylurea	5 (1.0)
	• Metformin + SGLT2 inhibitors	2 (0.4)
	• Insulin + SGLT2 inhibitors	1 (0.2)
	• Sulfonylure + SGLT2 inhibitors	1 (0.2)
	Three drug combination	95 (19.2)
	• Insulin + Metformin + Sulfonylurea	51 (10.3)
	• 2 insulin + Metformin	41 (8.3)
	• Sulfonylurea + Metformin + SGLT2 inhibitors	2 (0.4)
	• 2 insulin + SGLT2 inhibitors	1 (0.2)
	Four drug combination	7 (1.4)
	• 2 Insulin + Metformin + SGLT2 inhibitors	5 (1.0)
	• Insulin + Metformin + Sulfonylurea + SGLT2 inhibitors	2 (0.4)

Table 6 shows the amount of antihypertensive and lipid-lowering medication prescribed in the target population. The majority of patients were prescribed the Fixed Dose Combination (FDC) such as the FDC of Amlodipin and Perindopril (25.8%), the FDC of Amlodipin and Statin (19.2%), FDC of Telmisartan and Hydrochlorothiazide (18.3%). For lipid-lowering medication, most people (77.6%) were prescribed atorvastatin (alone or in combination), followed by rosuvastatin (20.7%).

**Table 6:** Antihypertensive and lipid-lowering medication prescribed in the population.

Categories	Medicines	n(%)
Antihypertensive drugs (n = 229)	**Monotherapy**	
	• Amlodipine alone	20 (8.7)
	• AT2 receptor antagonist (Losartan, Candesartan)	33 (14.4)
	• Beta blockers (Bisoprolol, Metoprolol)	21 (9.2)
	**Fixed dose combination (FDC)**	
	**Combination with Amlodipine**	
	• Amlodipine and Perindopril combination	59 (25.8)
	• Amlodipine and Statin combination	44 (19.2)
	• Amlodipine and Indapamide combination	3 (1.3)
	**Combination with agents acting on the renin-angiotensin system**	
	• FDC of Telmisartan and Hydrochlorothiazide	42 (18.3)
	• FDC of Valsartan and Hydrochlorothiazide	32 (14.0)
	• FDC of Candesartan and Hydrochlorothiazide	12 (5.2)
	• FDC of Enalapril and Hydrochlorothiazide	16 (7.0)
	• FDC of Lisinopril and Hydrochlorothiazide	15 (6.6)
	**FDC of Bisoprolol and Hydrochlorothiazide**	6 (2.6)
Lipid-lowering medication (n = 174)	Atorvastatine and Ezetimibe	81 (46.6)
	Atorvastatine and Amlodipine	44 (25.3)
	Atorvastatine	10 (5.7)
	Rosuvastatine	36 (20.7)
	Fenofibrate	3 (1.7)

### Drug-related problems

74.3% of patients had at least one DRP per prescription, while this number exhibited through the interview was 89.8% (Table 7). A total of 762 DRPs were identified through prescription reviews, and 412 DRPs were revealed through patient interviews. The mean DRP was 1.54±1.07 DRP and 2.62 ± 1.43 DRP per order and patient, respectively. Adverse drug reactions were the most frequent DRP in the study (68.8%). The majority of causes belonged to the patient, including “patient is unable to understand instructions properly or is not properly instructed”, “patient stores drug inappropriately”, “patient decides to use unnecessary drugs” and “patient intentionally uses or takes less drug than prescribed” accounting for 65.0%, 41.4%, 38.2%, and 28.7%, respectively. Looking at the prescribing, "inappropriate drug according to guidelines or formulary” and “no or incomplete drug treatment in spite of existing indication” accounted for the highest proportion with the rate of 45.0% and 42.9%, respectively. “Drug dose too low or too high”, and “Dosage regimen too frequent” were not found in the study (Table 8).

**Table 7:** Amount of DRP occurred in patients.

Number of DRP	Per prescription order (n, %)	Per patient interview (n, %)
0	127 (25.7)	16 (10.2)
1	60 (12.1)	18 (11.5)
2	232 (46.9)	34 (21.7)
3	66 (13.3)	43 (27.4)
4	10 (2.0)	35 (22.3)
5	0 (0.0)	9 (5.7)
6	0 (0.0)	2 (1.3)
**Sum**	**495 (100)**	**157 (100)**

**Table 8:** Type of drug-related problems among patients with type 2 diabetes population.

Code V9.1	Primary domains	Medication order review: n (%)	Patient adherence review: n (%)
P2.1	Adverse drug event (possibly) occurring	7 (0.9)	108 (68.8)
C1.1	Inappropriate drug according to guidelines/formulary	343 (45.0)	
C1.2	No indication of drug	3 (0.4)	
C1.3	Inappropriate combination of drugs, drugs and herbal medications, or drugs and dietary supplements	5 (0.7)	
C1.4	Inappropriate duplication of a therapeutic group or active ingredient	1 (0.1)	
C1.5	No or incomplete drug treatment in spite of existing indication	327 (42.9)	
C1.6	Too many different drugs/active ingredients prescribed for an indication	7 (0.9)	
C2.1	Inappropriate drug form/formulation (for this patient)	24 (3.1)	
C3.1	Drug dose too low	-	
C3.2	Drug dose of a single active ingredient too high	-	
C3.3	Dosage regimen is not frequent enough	33 (4.3)	
C3.4	Dosage regimen too frequent	-	
C3.5	Dose timing instructions wrong, unclear, or missing	12 (1.6)	
C7	**Patient related**		
C7.1	Patient intentionally uses/takes less drug than prescribed or does not take the drug at all for whatever reason		45 (28.7)
C7.2	Patient uses/takes more drugs than prescribed		7 (4.5)
C7.3	Patient abuses drug (unregulated overuse)		NA
C7.4	Patient decides to use unnecessary drugs		60 (38.2)
C7.5	Patient takes food that interacts		13 (8.3)
C7.6	Patient stores drugs inappropriately		65 (41.4)
C7.7	Inappropriate timing or dosing intervals		4 (2.5)
C7.8	Patient unintentionally administers/uses the drug in a wrong way		6 (3.8)
C7.9	Patient is physically unable to use the drug/form as directed		2 (1.3)
C7.10	Patient is unable to understand instructions properly or is not properly instructed		102 (65.0)
	**Total**	**762 (100)**	**412 (100)**
	**Mean±SD**	**1.54±1.07**	**2.62±1.43**

### Factors that were significantly associated with DRPs in T2DM patients

Age, duration of diabetes, polypharmacy, and cormobidity were independent predictors of DRPs. Patients with age over 60 were 3.38 times more likely to have DRPs than others (95% CI: 1.01–11.30; p = 0.048), and patients with a duration of diabetes of 10 years or more were 3.61 times more likely to have DRPs (95% CI: 1.11–11.74; p = 0.033). People with polypharmacy and cormobidity were 2.95 and 5.31 times more likely to have DRPs compared to those who had no polypharmacy or cormobidity (95% CI: 1.01–8.72, p = 0.049 and 95% CI: 1.97–14.30; p = 0.001, respectively) (Table 9). In patients, poor knowledge of antidiabetic agents was the main reason associated with adverse drug reactions (OR = 2.49; 95% CI: 1.54–4.03), while non-adherence was not significantly related to the occurring side effects of drugs (Table 10). Poor knowledge of antidiabetic agents is also significantly associated with non-adherence in patients (Table 11).

**Table 9:** Regression analysis of variables predicting DRPs among T2DM patients.

Variables	DRP		OR	CI 95%			p
	No	Yes					
Age over 60 years	103	293	3.38	1.01	-	11.30	0.048
Male sex	54	158	0.65	0.30	-	1.42	0.277
Duration of diabetes 10 years and more	4	77	3.61	1.11	-	11.74	0.033
Polypharmacy	65	83	2.95	1.01	-	8.72	0.049
Comorbidity	115	327	5.31	1.97	-	14.30	0.001

**Table 10:** Patient-related factors affect adverse drug events occurring.

Variables	ADR		OR	CI 95%			p
	No	Yes					
Adherence	Yex	22	38	1.24	0.76	-	2.03	0.39
	No	27	70					
Knowledge	Adequate	28	13	2.49	1.54	-	4.03	0.001
	Inadequate	21	95					

**Table 11:** Association between knowledge and adherence behavior of patients.

Variables		Adherence		OR	CI 95%			p
		No	Yes					
Knowledge	Adequate	18	23	2.73	1.32	-	5.66	0.007
	Inadequate	79	37					

## Discussion

Unsafe medication use and medication errors are leading causes of injury and avoidable harm all over the world. Reducing the level of severe, avoidable medication-related harm by 50% over 5 years globally was the goal of the Global Patient Safety Challenge 2017 [22]. Among the various types of medication errors, prescription errors showed the highest number of cases (51%) [23]. A medication review is a systematic assessment of a patient’s medication management to optimize the quality of the use of medicines and minimize medication-related problems [21]. The Australian National Safety and Quality Health Service Standards describe three types of medication reviews: i) Prescription or medication order review: a review of individual medication orders and/or prescription validity; ii) Concordance or medication adherence review: a review of a patient’s medicine-taking behavior, iii) Clinical medication review: a comprehensive review of a patient’s medicines in the context of their clinical conditions [21]. This article conducted a prescription review and patient adherence review to determine drug-related problems in prescribing and patient administration procedures.

T2DM is a chronic metabolic disorder that usually occurs in older people with comorbidity and polypharmacy. These are the factors that predicted a high risk of DRP. In this study, a total of 1174 DRPs were identified in both prescription reviews and patient adherence reviews. The mean DRP was 1.54 ± 1.07 DRP and 2.62 ± 1.43 DRP per order and patient review, respectively. Adverse drug reactions were the most frequent DRP in the study (68.8%). This result was similar to that in a systematic review conducted by Xiao-Feng Ni et al. (2021) [5]. Xiao-Feng Ni et al. reviewed 27 articles on DRPs in primary healthcare institutions all over the world and got results: the incidence of DRPs was reported in 14 studies and ranged from 8.54 to 99.16% (our report: 74.3% and 89.8% in prescription order and patient reviews, respectively), and the average number of DRPs per patient ranged from 0.58 to 7.2 (our study: 1.54 and 2.62 in prescriber and patient, respectively). The most common type of DRP reported by Xiao-Feng Ni was “treatment safety” (41.62%). A similar result was achieved in our study, where the percentage was 68.8%. Among the causes of DRPs in the article by Xiao-Feng Ni, the largest number belonged to drug selection, including C1.1 “inappropriate drug according to guidelines or formulary “(54.3%), C1.5”no or incomplete drug treatment in spite of an existing indication” (8.73%). The same was true in our study, with several 45.0%, and 42.9%, respectively. The number of DRP C1.5 in our study was higher than in the other studies, partly due to the breakdown of the drug supply at that time.

Besides, in the article by Xiao-Feng Ni [5], he pointed out that the second-most common reason that caused DRP was dose selection (C3), including “drug too low” and “drug too high”. In our study, we could not confirm these errors because, at the point of collection, no range of dose outside the normal range was observed in each medication among the whole prescription. To conclude the error of dose selection, we need to apply level 3 in the medication review process, which is clinical medication review, including a comprehensive review of a patient’s medicines in the context of their clinical conditions. The third cause of DRPs in a systematic review done by Xiao-Feng Ni was patient-related, with the common subdomain “patient intentionally uses or takes less drug than prescribed or does not take the drug at all for whatever reason” (1.22%). Other causes related to patients only accounted for a trivial proportion. However, in our study, a majority of patient-related DRPs were found, with the most frequency being "patient is unable to understand instructions properly or is not properly instructed", “patient stores insulin inappropriately”, “patient decides to use unnecessary drugs” and “patient intentionally uses or takes less drug than prescribed or did not take the drug at all for whatever reason”, which accounted for 65.0%, 41.4%, 38.2%, and 28.7%, respectively. The most common DRP in patients was "patient is unable to understand instructions properly or is not properly instructed", proving that health professionals need to spend more time with patients to explain the effects and side effects of drugs, the importance of medication adherence, the way to follow and assess ADR, how to use and store drugs, especially insulin, and special pharmaceutical forms like modified-released drugs, extended-release drugs, etc.

Patients’ knowledge and experiences play an important role in improving clinical outcomes. Alex H. Krist (2017) demonstrated that engaging patients in their care is essential to improving effectiveness, improving satisfaction with the care experience, reducing costs, and even benefiting the clinician’s experience [24]. In this study, the result from Table 11 indicated that poor knowledge of antidiabetic agents was significantly associated with adverse drug reactions (OR = 2.49; 95% CI: 1.54–4.03). Therefore, giving patients appropriate education is needed to improve clinical effectiveness and prevent potential ADRs. Besides, developing validation tools to recognize and assess DRP is necessary for clinical practices.

## Conclusion

The study was conducted on 495 diabetes mellitus outpatients in Thanh Nhan hospital in Vietnam and identified 1174 DRPs in both prescribing orders and patients’ interviews. There was a close association between age, duration of diabetes, polypharmacy, cormobidity, lack of knowledge, and the occurrence of DRPs. “Inadequate knowledge” was a main patient-related factor associated with DRPs, especially related to occurring adverse drug events. Interventions aim to improve the knowledge of patients, which is the clinical pharmacist’s cornerstone mission in the next period.

### Limitation

Since this study was cross-sectional rather than longitudinal, some indicators like "medication reconciliation problem" or "treatment effectiveness" have not been assessed. The overall appropriateness of the medications in a prescription has not yet been taken into account by this research. As a result, this requires considerable consensus among health professionals as well as time and human resources. Another drawback of the patient-agreed interview is that interviewers are only able to perform in a small sample size. Future work should address all of the aforementioned shortcomings and emphasize the connection between DRPs and therapeutic consequences. However, to increase the quality of prescribing as well as patient adherence to therapy, this study is able to identify several DRPs and associated variables that may be clinically addressed. Overall, the findings of this study cannot be generalized given the small size and the single-centered nature of this study.

## Supporting information

S1 Checklist (PDF)

S1 Appendix Certification approved by the ethics council in biomedical research and STROBE statement. (PDF)

## Abbreviation

DRPs: Drug-related Problems
ADR: Adverse Drug Reaction
PCNE: Pharmaceutical Care Network Europe
OR: odd ratio
CI: Confidence interval
T2DM: type 2 diabetes mellitus
CVD: cardiovascular disease
SGLT2: Sodium-glucose Cotransporter-2
FBG: fasting blood glucose
CHO: (Cholesterol)
LDL: (low-density lipoprotein)
TG: (triglyceride)
HDL: (high-density lipoprotein)
eGFR: (estimated Glomerular Filtration Rate)
