# Supplementary material for: Determination of drug-related problems among type 2 diabetes outpatients in a hospital in Vietnam: A cross-sectional study
Source: PLoS One. 2023 Aug 23;18(8):e0289825. doi: 10.1371/journal.pone.0289825 (PMC10446199; doi:10.1371/journal.pone.0289825)
Supplement: S1 Appendix — (PDF) [file pone.0289825.s002.pdf]

TRANSLATION

UNIVERSITY OF MEDICINE AND PHARMACY
VIETNAM NATIONAL UNIVERSITY, HANOI
ETHICS COMMITTEE
Number: …. /2022/CN-HĐĐĐ

SOCIALIST REPUBLIC OF VIETNAM
Independence - Freedom – Happiness

Hanoi, 09th December, 2022

CERTIFICATION
Approved by the Ethics Council in Biomedical Research

Pursuant to Circular No. 04/2020/TT-BYT dated March 5, 2020 of the Minister of Health regulations on the establishment, functions, tasks and powers of the Ethics Council in biomedical research;

Pursuant to the Decision No. 866/QD-ĐHYD dated September 15, 2021 of the Rector of the University of Medicine and Pharmacy on the establishment of the Ethics Council in biomedical research for the term 2019-2024;

Pursuant to the minutes of the first meeting No. 13/2022/BB-HĐĐĐ dated September 27, 2022 and the minutes of the second meeting No. 14/2022/BB-HĐĐĐ dated November 9, 2022 of the Ethics Council in biomedical research, University of Medicine and Pharmacy;

The Ethical Council in Biomedical Research at University of Medicine and Pharmacy, Vietnam National University, Hanoi with operational code IRB-VN 01016 (issued by Vietnam's Ministry of Health), and code IRB00013221 Vietnam National University, Hanoi University of Medicine and Pharmacy (issued by US Department HHS-OHRP) approved:

Consent on ethical and scientific aspects of research for the following:

1. Study title: ***Evaluation of drug-related problems among type 2 diabetes outpatients in Thanh Nhan Hospital***
2. Principal Investigator: *Assoc. Prof. Duong Thi Ly Huong*
3. Host organisation: Phenikaa University
- Recruitment location: Thanh Nhan Hospital, Hanoi
- Research subjects: patients aged 18 years and older, diagnosed with type 2 diabetes, assigned to outpatient treatment at Thanh Nhan hospital from October to December 2022, signed consent to participate in the study.
4. Expected number: 400 patients
5. Research time: 10/2022 - 10/2023
6. Sponsor: Phenikaa University

Approved documents include:

1. Research protocol, version No. 3.0 dated December 1, 2022
2. Information for research subjects and consent form to participate in the research (ICF), version 3.0 dated December 8, 2022

Approval date: December 9, 2022

Principal Investigator is responsible to:

- Comply with the protocol approved by the Ethics Council, Standard Operating Procedure, GCP principles, and current Vietnamese law provisions on ethics in biomedical research.
- Report to the Ethics Council in Biomedical Research at VNU University of Medicine and Pharmacy serious adverse events (SAE), research progress and the briefly of research results after the end of the study in accordance with current regulations
- Inform the Council of the termination of the study, the termination of the study before the expected completion date, the reason for the early termination./.

Recipient:

- Principal Investigator
- Storage: HĐĐĐ, L2.

CHAIRMAN

Assoc. Prof. Le Thi Luyen

TRƯỜNG ĐẠI HỌC Y DƯỢC -
ĐH QUỐC GIA HÀ NỘI
HỘI ĐỒNG ĐẠO ĐỨC
TRONG NGHIÊN CỨU Y SINH HỌC
Số: /2022/CN-HĐĐĐ

CỘNG HÒA XÃ HỘI CHỦ NGHĨA VIỆT NAM
Độc lập - Tự do - Hạnh phúc

Hà Nội, ngày 09 tháng 12 năm 2022

GIẤY CHỨNG NHẬN
Chấp thuận của Hội đồng đạo đức trong nghiên cứu y sinh học

Căn cứ Thông tư số 04/2020/TT-BYT ngày 05/03/2020 của Bộ trưởng Bộ Y tế về việc quy định việc thành lập, chức năng, nhiệm vụ, quyền hạn của Hội đồng đạo đức trong nghiên cứu y sinh học;

Căn cứ Quyết định số 866/QĐ-ĐHYD ngày 15/09/2021 của Hiệu trưởng Trường Đại học Y Dược về việc thành lập Hội đồng đạo đức trong nghiên cứu y sinh học Trường nhiệm kỳ 2019-2024;

Căn cứ Biên bản họp lần 1 số 13/2022/BB-HĐĐĐ ngày 27/09/2022 và Biên bản họp lần 2 số 14/2022/BB-HĐĐĐ ngày 09/11/2022 của Hội đồng đạo đức trong nghiên cứu y sinh học Trường Đại học Y Dược;

Hội đồng đạo đức trong nghiên cứu y sinh học Trường Đại học Y Dược ĐH Quốc gai Hà Nội có mã số hoạt động IRB-VN 01016 (Bộ Y tế Việt Nam cấp), IRB00013221 Vietnam National University, Hanoi University of Medicine and Pharmacy (U.S. Department HHS – OHRP cấp) chấp thuận:

Chấp thuận về các khía cạnh đạo đức và khoa học trong nghiên cứu đối với nghiên cứu sau:

1. Tên đề tài: ***Phân tích các vấn đề liên quan đến sử dụng thuốc điều trị đái tháo đường ở bệnh nhân ngoại trú Bệnh viện Thanh Nhàn***
2. Chủ nhiệm đề tài: *PGS.TS. Dương Thị Ly Hương*
3. Cơ quan chủ trì: Trường Đại học Phenikaa

- Địa điểm tuyển chọn: Bệnh viện Thanh Nhàn, Hà Nội
- Đối tượng nghiên cứu:
    - Bệnh nhân từ 18 tuổi trở lên, chẩn đoán đái tháo đường typ II, được chỉ định điều trị ngoại trú tại BV Thanh Nhàn từ tháng 10 – tháng 12 năm 2022, ký chấp thuận tham gia nghiên cứu

4. Số lượng đối tượng dự kiến: 400 bệnh nhân
5. Thời gian nghiên cứu: 10/2022 – 10/2023

Được quét bằng CamScanner

6. Đơn vị tài trợ nghiên cứu: Trường Đại học Phenikaa

Các tài liệu được chấp thuận bao gồm:

1. Đề cương nghiên cứu phiên bản số 3.0 ngày 01/12/2022.
2. Phiếu thông tin cho đối tượng nghiên cứu và chấp thuận tham gia nghiên cứu (ICF), phiên bản số 3.0 ngày 8/12/2022.

Ngày chấp thuận: *Ngày 09 tháng 12 năm 2022*

Chủ nhiệm đề tài có trách nhiệm

- Tuân thủ đề cương nghiên cứu đã được Hội đồng đạo đức trong nghiên cứu Y sinh học chấp thuận, các quy trình thực hiện chuẩn, các nguyên tắc GCP, các quy định của pháp luật Việt Nam hiện hành về đạo đức trong nghiên cứu y sinh học.
- Báo cáo Hội đồng đạo đức trong nghiên cứu y sinh học Trường Đại học Y Dược ĐHQGHN các biến cố bất lợi nghiêm trọng (SAE), tiến độ nghiên cứu và bản tóm tắt kết quả nghiên cứu sau khi kết thúc nghiên cứu theo đúng các quy định hiện hành.
- Báo cáo Hội đồng đạo đức trong nghiên cứu y sinh học Trường Đại học Y Dược ĐHQGHN để xem xét và chấp thuận những thay đổi, hay chỉnh sửa bất kỳ trong đề cương nghiên cứu, thông tin trong ICF và các tài liệu khác liên quan đến cung cấp thông tin cho đối tượng tham gia nghiên cứu trước khi áp dụng trong nghiên cứu.
- Thông báo cho Hội đồng về việc ngừng nghiên cứu, kết thúc nghiên cứu trước thời hạn hoàn thành dự kiến, lý do của việc kết thúc sớm./.

Nơi nhận:
- Chủ nhiệm đề tài;
- Lưu HĐĐĐ, L2.

CHỦ TỊCH

PGS.TS. Lê Thị Luyến

Được quét bằng CamScanner

TRANSLATION

INFORMATION FOR RESEARCH SUBJECTS AND CONSENT FORM TO PARTICIPATE IN THE RESEARCH

Study title: Evaluation of drug-related problems among type 2 diabetes outpatients in Thanh Nhan Hospital

Principal Investigator: Assoc. Prof. Duong Thi Ly Huong

Host organisation: Phenikaa University

Partner organisation: Thanh Nhan Hospital, Hanoi

Sponsor: Phenikaa University

I. INFORMATION OF THE RESEARCH

Purpose and conduct of the research

- ***Why is the research conducted?***

The study is conducted to collect problems related to drug use and analyze the factors affecting drug use. On the basis of which, interventions are proposed aim to reduce the drug-related problems, enhance treatment efficacy, unwanted effects, costs and disease burden for patients and society.

- ***How will the research be conducted? duration of the study, inclusion and exclusion criteria, and the number of people who will participate in the study.***

The research take place over a year, including the process of ideation, proposal writing and research report. The study participants are diabetic patients with medical records managed at Thanh Nhan hospital's examination department. Not any interfere to the patient's medical examination and treatment process, the researchers only collect data from the medical records and ask participants a questionaire related to knowledge and understanding of the drug and drug use, as well as their behavior of adherence to prescribed medication. Specifically, the investigator will interview participants the questionaire about: how to take antidiabetic medications (oral and parenteral), difficulties in taking prescribed medications, side effects when taking medications, the doctor's or pharmacist's consultants and patient adherence. Each patient will be interviewed one time. At the same time, the researcher also collects information from the medical record about: medical history, biochemical laboratory results related to the disease, information in the prescriptions.

The number of patients participating in the study: at least 400

- ***What is the nature and extent of participation of the study participants?***

The study participants do not have to undergo any physical intervention, only take a little time to answer the questionair (about 10-15 minutes). The interview process can be recorded. The information is used for research only, not used for any other purposes.

The risks and disadvantages

- ***Are there any risks to participants? Detailed description***

This is a non-interventional study, not any physical risk to participants can be happened. During the interview process, if a problem is detected that requires intervention, the research team will notify the clinical pharmacist for timely intervention.

- ***Are there any other impacts that participants need to know before deciding to participate in the study?***

There are not any impacts affect to the patients, or the treatment process of patients.

Possible benefits for participants

- ***What benefits can participants expect?***

The results of the study are recommendations to improve treatment efficiency, minimize side effects, reduce costs and disease burden for patients.

Cost/Pay for participants

- ***What will be paid in the study?***

This is an epidemiological investigation study. Participants join the study don't have to pay an extra costs, nor don't receive any fees for participating in the interview.

- ***Will travel expenses be reimbursed or not, how much in detail? Is there compensation for the loss of income? Daily cost of meals?***

The research does not affect the patient's life, medical examination and treatment, therefore there is no funding payment or reimbursement.

- ***What is the method of payment?***

No application

Compensation/treatment for patients when have research-related injury

- ***Do participants receive free treatment in the case of injury occuring caused by participating in the study?***

The study did not affect the physical health of the study participants, so this does not apply.

- ***Do participants receive free treatment in the case of injury occuring caused by non-compliance with the study?***

The study does not require participants' compliance with the study protocol, so this does not apply.

Contact

Assoc. Prof. Duong Thi Ly Huong, phone number: 0395282456

Voluntary participation

- Participants have the right to decide for themselves, not forced to participate
- Participants can withdraw at any time without affecting the treatment/care they are receiving.

Security

- Participants' identities are encrypted to ensure confidentiality in biomedical research. Only researchers are allowed access to research participants' information. The research data is for scientific purposes only and does not serve any other purpose.

II. AGREEMENT TO PARTICIPATE IN THE STUDY

I have read and understand the information above, have had the opportunity to review and ask information related to the content of the study. I spoke directly to the investigator and all questions are answered satisfactorily. I receive a copy of the Information Statement For Research Subjects and Consent to Participate in this study. I voluntarily agree to participate.

Participant's signature:

Signature ___________________

Full name ____________________ Date _________________

Investigator/consentee's signature:

I, the undersigned, certify that the patient/volunteer who signed the informed consent form has read the totally above information, which has been fully explained to him/her and he/she has fully understood the nature, risks and benefits of participation in the study.

Signature ______________

Full name ____________________ Date _________________

BẢN THÔNG TIN DÀNH CHO ĐỐI TƯỢNG NGHIÊN CỨU VÀ CHẤP THUẬN THAM GIA NGHIÊN CỨU

Tên nghiên cứu: Phân tích các vấn đề liên quan đến sử dụng thuốc điều trị đái tháo đường ở bệnh nhân ngoại trú Bệnh viện Thanh Nhàn
Nghiên cứu viên chính: PGS.TS. Dương Thị Ly Hương
Đơn vị chủ trì: Trường Đại học Phenikaa
Đơn vị phối hợp: Bệnh viện Thanh Nhàn
Nhà tài trợ: Trường Đại học Phenikaa

I.THÔNG TIN VỀ NGHIÊN CỨU

Mục đích và tiến hành nghiên cứu

- ***Vì sao nghiên cứu được tiến hành?***
  Nghiên cứu được tiến hành nhằm thu thập các vấn đề liên quan đến sử dụng thuốc và phân tích các yếu tố ảnh hưởng đến sử dụng thuốc, trên cơ sở đó đề xuất các biện pháp can thiệp nhằm giảm thiểu vấn đề liên quan đến sử dụng thuốc, nâng cao hiệu quả điều trị, giảm tác dụng không mong muốn, giảm chi phí và gánh nặng bệnh tật cho bệnh nhân và xã hội.
- ***Nghiên cứu sẽ được tiến hành như thế nào? khoảng thời gian tiến hành, tiêu chuẩn lựa chọn và loại trừ, số người sẽ tham gia vào nghiên cứu.***
  Nghiên cứu được diễn ra trong 1 năm, bao gồm cả quá trình lên ý tưởng, viết đề cương và báo cáo nghiên cứu. Người tham gia nghiên cứu là bệnh nhân đái tháo đường, có hồ sơ quản lý tại khoa khám bệnh bệnh viện Thanh Nhàn. Nghiên cứu không can thiệp vào quá trình khám chữa bệnh của bệnh nhân. Các nghiên cứu viên chỉ thu thập số liệu từ bệnh án nghiên cứu và hỏi bệnh nhân một số câu hỏi liên quan đến kiến thức và hiểu biết của bệnh nhân về thuốc và sử dụng thuốc, cũng như hành vi tuân thủ dùng thuốc theo đơn của bệnh nhân tiểu đường. Cụ thể, nghiên cứu viên sẽ hỏi bệnh nhân những câu hỏi có sẵn về: cách dùng thuốc chống đái tháo đường (đường uống và đường tiêm), những khó khăn khi dùng thuốc trong đơn, các tác dụng phụ khi dùng thuốc, việc tư vấn sử dụng thuốc của các bác sĩ, dược sĩ, việc tuân thủ dùng thuốc của bệnh nhân. Mỗi bệnh nhân sẽ được phỏng vấn 1 lần. Đồng thời, nghiên cứu viên cũng thu thập các thông tin từ bệnh án về: tiền sử bệnh, các kết quả xét nghiệm sinh hóa liên quan đến bệnh, các thông tin về đơn thuốc.
  Số bệnh nhân tham gia vào nghiên cứu: ít nhất 400
- ***Bản chất và mức độ tham gia của những người tham gia nghiên cứu là gì?***
  Người tham gia nghiên cứu không phải chịu bất cứ một can thiệp gì về mặt thể chất, chỉ mất chút thời gian để tham gia trả lời phỏng vấn của nghiên cứu viên (khoảng 10-15 phút). Quá trình phỏng vấn có thể được ghi âm. Thông tin chỉ phục vụ cho nghiên cứu mà không được dùng cho bất kỳ mục đích nào khác.

Các nguy cơ và bất lợi

- ***Liệu có những nguy cơ nào? Mô tả chi tiết***
  Nghiên cứu không can thiệp nên không có bất kỳ một nguy cơ nào về mặt thể chất đối với người tham gia nghiên cứu. Trong quá trình phỏng vấn, nếu phát hiện thấy có vấn đề cần can thiệp, nhóm nghiên cứu sẽ thông báo với dược sĩ lâm sàng biết để có các can thiệp kịp thời.
- ***Có những tác động khác mà người tham gia cần biết khi quyết định tham gia nghiên cứu?***
  Không có bất kỳ tác động nào ảnh hưởng đến người bệnh, hoặc quá trình điều trị bệnh.

Những lợi ích có thể có đối với người tham gia

- ***Những người tham gia có thể mong đợi những lợi ích gì?***
  Kết quả của nghiên cứu là những đề xuất để nâng cao hiệu quả điều trị, giảm thiểu tác dụng không mong muốn, giảm chi phí và gánh nặng bệnh tật cho bệnh nhân.

Chi phí/chi trả cho đối tượng

- ***Những khoản sẽ được chi trả trong nghiên cứu***
  Đây là nghiên cứu điều tra dịch tễ học. Người tham gia nghiên cứu không phải trả thêm chi phí gì, và cũng không được nhận các khoản chi phí nào cho việc tham gia trả lời phỏng vấn.
- ***Chi phí đi lại có được bồi hoàn hay không, số lượng cụ thể? Có bù đắp cho việc mất thu nhập không? Chi phí ăn uống thường ngày?***
  Nghiên cứu không ảnh hưởng đến quá trình sinh sống, khám chữa bệnh của bệnh nhân nên không có khoản chi trả kinh phí cũng như bồi hoàn kinh phí.
- ***Hình thức và phương thức chi trả như thế nào?***
  Không

Bồi thường/điều trị khi có tổn thương liên quan đến nghiên cứu:

- ***Người tham gia có được điều trị miễn phí trong trường hợp xảy ra chấn thương hoặc tổn thương do việc tham gia vào nghiên cứu gây ra?***
  Nghiên cứu không ảnh hưởng đến sức khỏe thể chất của người tham gia nghiên cứu nên không áp dụng điều này.
- ***Người tham gia có được điều trị miễn phí trong trường hợp xảy ra tổn hại sức khỏe do việc không tuân thủ nghiên cứu gây ra?***
  Nghiên cứu không đòi hỏi về sự tuân thủ của người tham gia đối với đề cương nghiên cứu nên không áp dụng điều này.

Người liên hệ

PGS.TS. Dương Thị Ly Hương, số điện thoại: 0395282456

Sự tự nguyện tham gia

- Người tham gia được quyền tự quyết định, không hề bị ép buộc tham gia
- Người tham gia có thể rút lui ở bất kỳ thời điểm nào mà không bị ảnh hưởng gì đến việc điều trị/chăm sóc mà họ đáng được hưởng.

Tính bảo mật

- Danh tính của người tham gia nghiên cứu được mã hóa để đảm bảo tính bảo mật trong nghiên cứu y sinh học. Chỉ nghiên cứu viên được phép tiếp cận với các thông tin của người tham gia nghiên cứu. Các số liệu nghiên cứu chỉ phục vụ mục đích khoa học mà không phục vụ bất cứ mục đích nào khác.

II. CHẤP THUẬN THAM GIA NGHIÊN CỨU

Tôi đã đọc và hiểu thông tin trên đây, đã có cơ hội xem xét và đặt câu hỏi về thông tin liên quan đến nội dung trong nghiên cứu này. Tôi đã nói chuyện trực tiếp với nghiên cứu viên và được trả lời thỏa đáng tất cả các câu hỏi. Tôi nhận một bản sao của Bản Thông tin cho đối tượng nghiên cứu và chấp thuận tham gia nghiên cứu này. Tôi tự nguyện đồng ý tham gia.

Chữ ký của người tham gia:

Chữ ký ____________________

Họ tên_____________________ Ngày tháng năm __________________

Chữ ký của Nghiên cứu viên/người lấy chấp thuận:

Tôi, người ký tên dưới đây, xác nhận rằng bệnh nhân/người tình nguyện tham gia nghiên cứu ký bản chấp thuận đã đọc toàn bộ bản thông tin trên đây, các thông tin này đã được giải thích cặn kẽ cho Ông/Bà và Ông/Bà đã hiểu rõ bản chất, các nguy cơ và lợi ích của việc Ông/Bà tham gia vào nghiên cứu này.

Chữ ký ____________________

Họ tên: Ngày tháng năm__________________
